# Theoretical Study on the Aggregation of Copper Clusters on a Liquid Surface

**DOI:** 10.3390/ma12233877

**Published:** 2019-11-24

**Authors:** Hong-Ying Mao, Bao-Xing Li, Wang-Feng Ding, Yu-Hong Zhu, Xu-Xin Yang, Chao-Yang Li, Gao-Xiang Ye

**Affiliations:** 1 Department of Physics, Hangzhou Normal University, Hangzhou 310036, China; dingwangfeng@126.com (W.-F.D.); zyh@hznu.edu.cn (Y.-H.Z.); youngxuxin@163.com (X.-X.Y.); zjlcy@163.com (C.-Y.L.); 2 Department of Physics, Zhejiang University, Hangzhou 310027, China; gxye@zju.edu.cn

**Keywords:** Cu clusters, aggregation, growth mechanism, simulation

## Abstract

The ground state structures of copper clusters with different sizes along with their aggregation have been systematic investigated using Amsterdam Density Functional (ADF) and Atomistix ToolKit (ATK) programs. On the basis of geometry optimization, some Cu clusters with more stable structures which were not reported previously have been revealed. In most cases, these Cu clusters prefer to adopt icosahedral structures which originate from the 13-atom icosahedron. It has also been demonstrated that the interaction between two Cu clusters is anisotropic, which is attributed to their charge distribution, especially the highest occupied molecular orbital (HOMO) and lowest unoccupied molecular orbital (LUMO) of Cu clusters. Moreover, we have carried out the simulation of Cu clusters aggregation on the silicone oil substrate by means of Monte Carlo (MC) method, which shows good consistence with our previous experimental studies.

## 1. Introduction

There are extensive research efforts for the investigation of metal clusters, which have been regarded as promising candidates for biomedical applications [1,2], chemical and biological testing [3], plasmonic sensitization [4] and optoelectronic applications [5]. Their unique physical and chemical properties mainly originated from their specific geometrical and micro-structures, leading to a large surface area-to-volume ratio. Versatile functionalities of metal clusters have been demonstrated by modulating their compositions. Among all kinds of metal clusters, copper clusters have been intensively studied in the last decade, including the aggregation geometry, electronic structures, physical and chemical properties [6,7,8]. Recently, the conversion efficiency of methanol from carbon dioxide over size-selected Cu_4_ clusters at low pressures has been investigated [9]. It was indicated that size-selected Cu_4_ clusters were the most active low-pressure catalyst for catalytic CO_2_ conversion to CH_3_OH. The size effect of copper cluster in methanol synthesis has also been investigated using in situ grazing incidence X-ray absorption spectroscopy, catalytic activity measurements and first-principles calculations [10]. It has been demonstrated that the activities of gas-phase Cu clusters increased as the cluster size decreased. Moreover, different theoretic methods, including self-consistent one-electron local-density theory, density functional calculations, Embedded-Atoms Method (EAM) and the Monte Carlo (MC) method, have been employed to investigate the size effect of metal clusters on their structural and electronic properties [11,12,13,14,15,16,17,18,19,20,21,22].

It was generally believed that the growth behavior of metal atoms on liquid substrate differed from that on solid substrate. Comparing with solid substrates, the interaction between metal atoms and liquid substrate is much weaker. The diffusion coefficient is much larger on liquid substrates than that on solid substrates. Moreover, because of the non-lattice characteristic of liquid substrates, liquid substrates can be regarded as an isotropic substrate. In our previous studies, Cu thin film on liquid substrate (silicone oil) has been fabricated using vapor phase deposition method [23]. Compact and ramified Cu clusters can be observed at the initial stage of growth. With the increasing thickness of Cu, the random diffusion of Cu clusters was found and larger ramified aggregates were observed on the silicone oil surface. Other metal nanoparticles and thin films have also been fabricated on liquid substrates, including Au, Ag, Al and Zn [24,25,26,27,28]. In spite of extensive researches for the morphology of metal nanostructures on liquid substrates, the aggregation of metal nanostructures was only discussed and analyzed by classical kinetic method and the growth mechanism of Cu nanostructures on liquid substrates remains unknown. In order to reveal the growth mechanism of Cu nanostructures, especially at the initial stage of growth, the investigation of fundamental structural characteristics of Cu clusters and their aggregation behaviors are highly desirable.

In the present study, first-principles calculations have been carried out to investigate the aggregation of Cu clusters on silicone oil at the initial growth stage using the ADF and ATK program, respectively. Cu clusters with different sizes have been studied. It has been demonstrated that Cu clusters prefer to adopt icosahedral structures deriving from the 13-atom icosahedron. Our calculation results also reveal an anisotropic interaction between two Cu clusters, which is attributed to the charge distribution of Cu clusters. Moreover, the aggregation of Cu clusters on the silicone oil substrate has been simulated by MC method which is in good agreement with our previous experimental studies.

## 2. Materials and Methods

In the ADF program version (2010.02) [29], molecular orbitals (MOs) were expanded using a large, unconstructed set of Slater-type orbitals (SOs)—Triple-zeta with two polarization functions (TZ2P) [30]. The TZ2P basis is an all-electron basis of triple-z quality, which is augmented by two sets of polarization functions. During the simulation, the frozen-core approximation for inner-core electrons was utilized. The copper orbital which is inner than Cu 3p were kept frozen. We use an auxiliary set of s, p, d, f and g SOs to fit the molecular density and to represent the Coulomb and exchange potentials in each self-consistent field (SCF) cycle. All calculations were made in the framework of the generalized gradient approximation (GGA) using the Becke-Perdew functional, which is based on Becket [31,32] gradient correction to the local expression for the exchange energy and the correlation energy. The self-consistent field was converged to a value of 10^−4^ in our studies.

Atomism Tool Kit (ATK) is a software package [33,34,35] that offers very fast geometry optimization and molecular dynamics calculations by using such methods ranging from both accurate quantum-mechanical first-principles and fast semi-empirical methods to classical potentials. Therefore, the software can treat large-scale systems with several thousand atoms. In this paper, the optimization of cluster structures has been made by using Perdew-Burke-Ernzerhof (PBE) functional within density functional theory (DFT) in generalized gradient approximation (GGA).

Kohn-Sham density functional theory has been widely used to simulate the ground state structure of metal clusters. In generalized gradient approximation (GGA), the general expression of the exchange-correlation density functional is described as:

(1) $$E_{xc}[n_{↑},n_{↓}]=\int d^{3}rf(n_{↑},n_{↓},\nabla n_{↑},\nabla n_{↓})$$

It was found that semiempirical GGA’s can be remarkably successful for small molecules. A first-principles numerical GGA can be constructed in the following ways. In a system of slowly varying density, the second-order density-gradient was first expanded for the exchange-correlation hole surrounding the electron. After that, its spurious long-range parts were cut off to satisfy sum rules on the exact hole. Perdew et al. improved the exchange-correlation density functional with the optimized Perdew-Burke-Ernzerhof (PBE) functional which is in the form of [35]:

(2) $$E_{{}_{xc}}^{GGA}[n_{↑},n_{↓}]=\int d^{3}rn[\varepsilon_{c}(r_{s},\xi )+H(r_{s},\xi ,t)]$$

where *r_s_* is the local Seitz radius ($n=3/4\pi r_{s}^{3}=k_{F}^{3}/3\pi^{2}$), $\xi =(n_{↑}-n_{↓})/n$ is the relative spin polarization and $t=|\nabla n|/2\varphi k_{s}n$ is a dimensionless density gradient. Here $\varphi (\xi )=[{\left(1+\xi \right)}^{2/3}+{\left(1-\xi \right)}^{2/3}]/2$ is a spin-scaling factor and $k_{s}=\sqrt{4k_{F}/\pi a_{0}}$ is the Thomas- Fermi screening wave number ($a_{0}=\hbar^{2}/me^{2}$). They constructed the gradient contribution H from three conditions, which were in the slowly and rapidly varying limits and under uniform scaling to the high-density limit, respectively. The atomization energies of small molecules obtained by PBE functional were in good agreement with the experimental results.

As we know, copper is a non-magnetic material. Except for some special doped structures [36,37,38], the pure copper clusters do not exhibit any nontrivial magnetic moments. ADF and ATK programs had been used to study the magnetic properties of small Cun (n ≤ 10) clusters and no additional magnetic moments were found. Therefore, spin polarization is not considered in the calculations. In order to simulate the aggregation of the Cu clusters on a liquid surface, a Cu cluster can be regarded as a Brownian particle and the forces exerting on it by liquid substrates are sum up as a random force. For a free cluster floating on the liquid substrate, its motion is mainly driven by Brownian dynamics. In this case, MC methods with small trial steps have been employed to simulate random Brownian motions. The simulations are initiated with 2000 clusters randomly distributed in the 2-dimensional system and are carried out in 3 steps. Firstly, MC steps are performed until the energy of system is optimized to a minimum. Then, another 2000 clusters are dispersed to the free space of the system one by one, following a random walk until it is attached firmly to aggregations of early clusters. Finally, the optimization of the total amount of clusters is performed to reach an energy minimum of the whole system.

## 3. Results

### 3.1. Copper Clusters Cu_n_ (n = 2–80)

We have investigated copper clusters with different sizes using the ADF program and the ATK software package, respectively. Initial atomic configurations are randomly placed within a real three dimensional box or cage or sphere. To avoid over-crowded or over-relaxed among atoms, their separations are confined to be 2.2–2.8 Å. For each Cu cluster with less than 32 Cu atoms (n ≤ 32), about one hundred thousand initial geometrical configurations have been constructed by this method. After that, the initial configurations are optimized using the ADF program and the ATK software package, respectively. Our results show that most initial structures are convergent after geometry optimization. In many cases, the same convergent structure is obtained after the geometry optimization of different initial configurations. At last, optimized structures with larger binding energies are optimized further with higher calculation accuracy. During the optimization, some isomers with different structures have almost the same binding energy. The cutoff energy is selected as 0.1 eV. Their energy order will not be reversed under different calculation accuracy when the energy difference between two isomers is larger than 0.1 eV. During structural optimization, the symmetries are not restricted. The atom vibration frequencies for each structure have also been calculated and structures with imaginary frequencies have been eliminated. As a result, if the energy difference between the structures is less than 0.1 eV, they are considered to be degenerated

Our calculations results of Cu_n_ (*n* = 2–80) constructed by Ref. [13,14] are presented in Table 1. The estimated binding energies of Cu clusters are different when the ADF or ATK program is employed but the trend of binding energies is similar. Although Jahn–Teller effect was mostly encountered in octahedral complexes of the transition metals and this phenomenon was commonly observed in six-coordinate copper(II) complexes. Since we are mainly focused on the formation of Cu clusters and their aggregations, Jahn-Teller effect is not considered in the present study.

**Figure 1:**
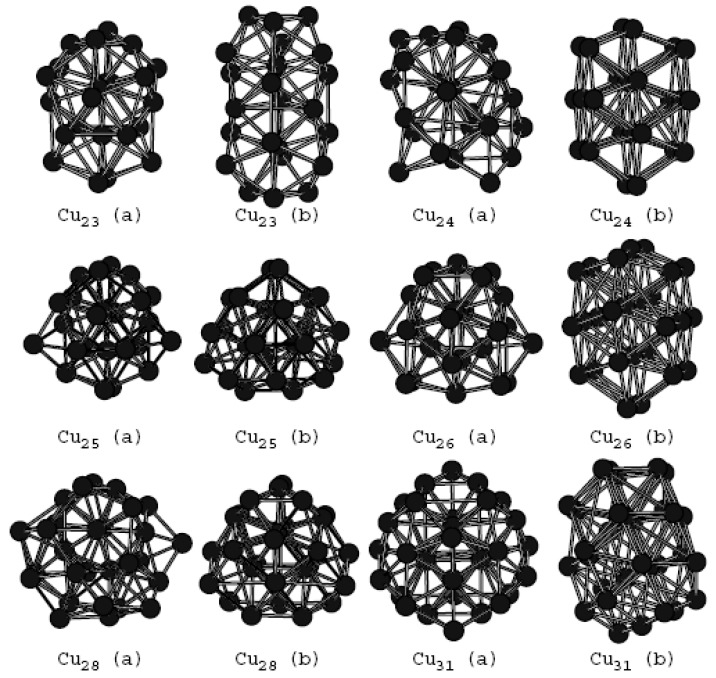
The newly obtained ground state structure (marked with “a”) except for *n* = 23 and *n* = 28 and the original ground state structure (marked with “b”).

In Table 1, the data marked with * in the range of n from 3 to 8 are calculated from experimental data [19,39]. The binding energy of Cu clusters calculated using ADF shows good consistence with experimental data. There is a discrepancy between the calculated binding energy using the ATK program and experimental data, which is attributed to the fact that DFT-GGA (density functional theory and generalized gradient approximation) does not capture long-range type electrostatic effects [40,41,42]. Choosing different methods or correlation functions based on first-principles calculations, the binding energies are different. But their change trends are nearly the same, which will be discussed later. Our calculation results indicate that the constructed Cu clusters are stable because they do not have imaginary frequencies. Furthermore, the global minima of Cu clusters ranging from 2 to 32 atoms have been investigated. Some Cu clusters with more stable structures, which have been not reported previously, are revealed. Geometric structures and binding energies of these Cu clusters are presented in Table 1 and Figure 1, respectively. The newly revealed structures are marked by the letter “a”. The letter “b” is used to refer to the structures in the previous study [14]. 

The Cu_31_(a) and Cu_31_(b) structure shown in Figure 1 are different but they are almost degenerated. Other structures marked with “a” are more stable than those marked with “b”. Cu_23_(a) and Cu_28_(a) structures are the same as those reported in Ref [19]. The most stable structure of the Cu_23_ cluster is the polyicosahedral structure in the form of a “triple icosahedron”. In the case of Cu_28_ cluster, it is a disordered structure with polycapped atoms by removing two surface atoms from the 19- atom triple icosahedron. All other structures marked with “a” are newly revealed but they are also based on the icosahedral growth. Although the energy difference between “a” and “b” structures calculated by the ADF program is different from those calculated by the ATK software package, the energy order between them does not change. In Figure 1, structure “a” is more stable than structure “b”.

**Figure 2:**
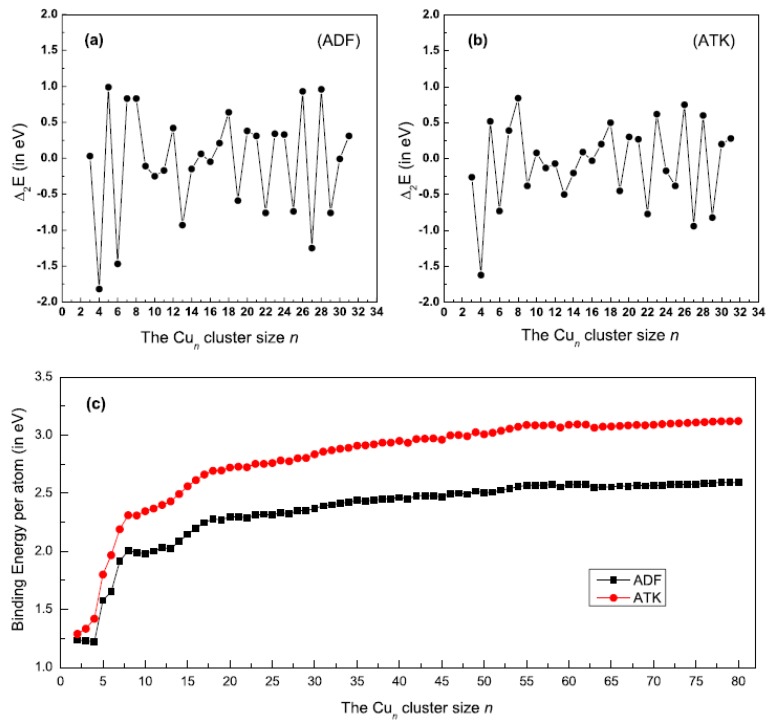
The second order difference (Δ_2_E) of the binding energies for the ground state structures of Cu_n_ (*n* = 2–32) clusters obtained by the (**a**) ADF and (**b**) ATK software, respectively; (**c**) The binding energy per atom as a function of Cu clusters with different sizes.

In order to have a better understanding about the structural evolution, we have also calculated the second order difference (Δ_2_E) of the binding energies for ground state structures of Cu_n_ (*n* = 2–32) clusters. The Δ_2_E function is defined as Δ_2_E = E(Cu_n − 1_) + E(Cu_n + 1_) − 2E(Cu_n_). The larger positive Δ_2_E’s demonstrated that the corresponding clusters (*n* = 5, 8, 18, 20, 26, 28) are more stable than their neighboring Cu clusters. The calculated results are shown in Figure 2a,b. Figure 2c presents the change of the binding energy per atom with the increasing number of Cu atoms. Comparisons between the ADF and ATK results show that they have similar trends. 

### 3.2. Aggregation of the Cun Clusters

**Figure 3:**
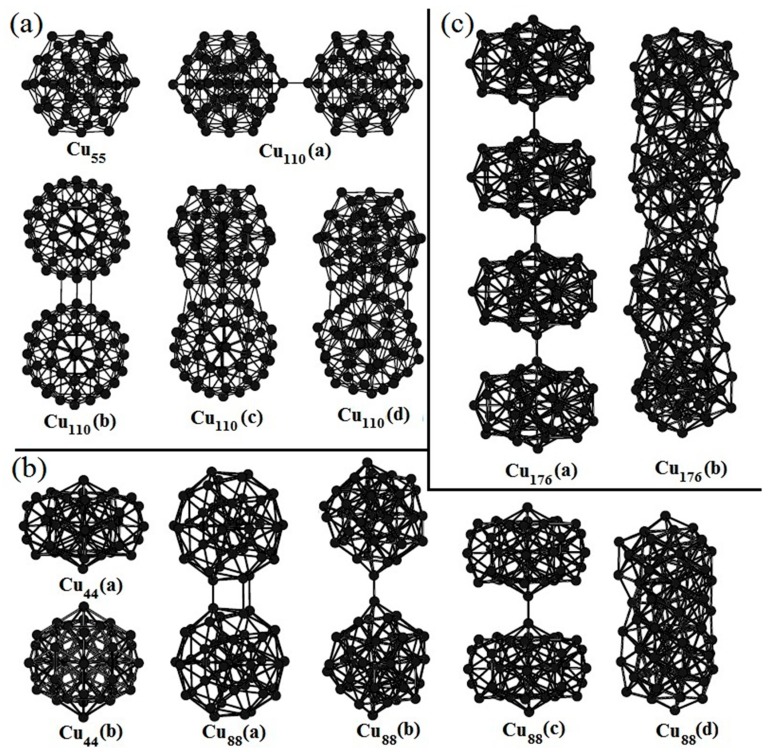
The aggregation of two typical (**a**) Cu_44_, (**b**) Cu_55_ and (**c**) four Cu_44_ clusters.

To date, there have been many reports about copper clusters but there is little investigation about the aggregation of Cu clusters. By means of the ATK software, we have studied the aggregation of medium-sized copper clusters. The optimized compact Cu_55_ in Figure 3a has an icosahedral structure which is its ground state structure. If two Cu_55_ structures approach to each other in different directions, it is found that the interaction between them is anisotropic, which means that their interaction is rather weak in some directions, while quite strong in other directions. Cu_110_(a) is the aggregation of two Cu_55_ clusters in the direction with relatively weak interaction. The binding energy between two Cu_55_ structures is only 0.96 eV. If they are approached in the direction with strong interaction, structural optimization reveals that they interact strongly with each other and reconstruct to form a new stable structure. The relaxed Cu_110_(b) in Figure 3a is its initial structure. Cu_110_(c) is its intermediate structure which is optimized to its final stable structure of Cu_110_(d). The energy released from the Cu_110_(b) to the Cu_110_(d) is up to 7.5 eV, which is much larger than that of Cu_110_(a).

The initial structure of Cu_44_(a) in Figure 3b is also a compact structure. After structural optimization, it becomes more compact which is denoted by Cu_44_(b). But the binding energy is 2.44 eV smaller than the ground state structure with 44 copper atoms (not shown here). Similar to the aggregation of two Cu_55_ clusters, the aggregation of two or more Cu_44_ is also highly dependent on the direction along which they are approached. After the geometry optimization of the initial Cu_88_(a), one stable structure Cu_88_(b) is obtained. The binding energy of the Cu_88_(b) is only 1.00 eV. If two Cu_44_(a) structures are approached in another direction (Cu_88_(c) in Figure 3b), a stable structure of Cu_88_(d) with larger binding energy of 14.89 eV is obtained. It is worth noting that the Cu_88_(d) is even more stable than the aggregated structures from the ground state structure with 44 copper atoms. A large number of calculations indicate that the maximum ratio of the binding energies between two medium-sized ground state structures in different directions is ranging from 7 to 15. In addition, the initial structure of Cu_176_(a) shown in Figure 3c is constructed from four Cu_44_(a) clusters. After geometry optimization, a completely different structure, Cu_176_(b), has been obtained. The reconstructed structure is highly stable with the binding energy of 32.56 eV. We need to mention that a large number of initial structures can be constructed using four Cu_44_(a) structures. The optimized structures are also different with each other. The aggregation of these clusters depends on the initial structures but quasi-linear stacking seems to be more favorable.

### 3.3. Anisotropic Interaction Between Two Cu Clusters

**Figure 4:**
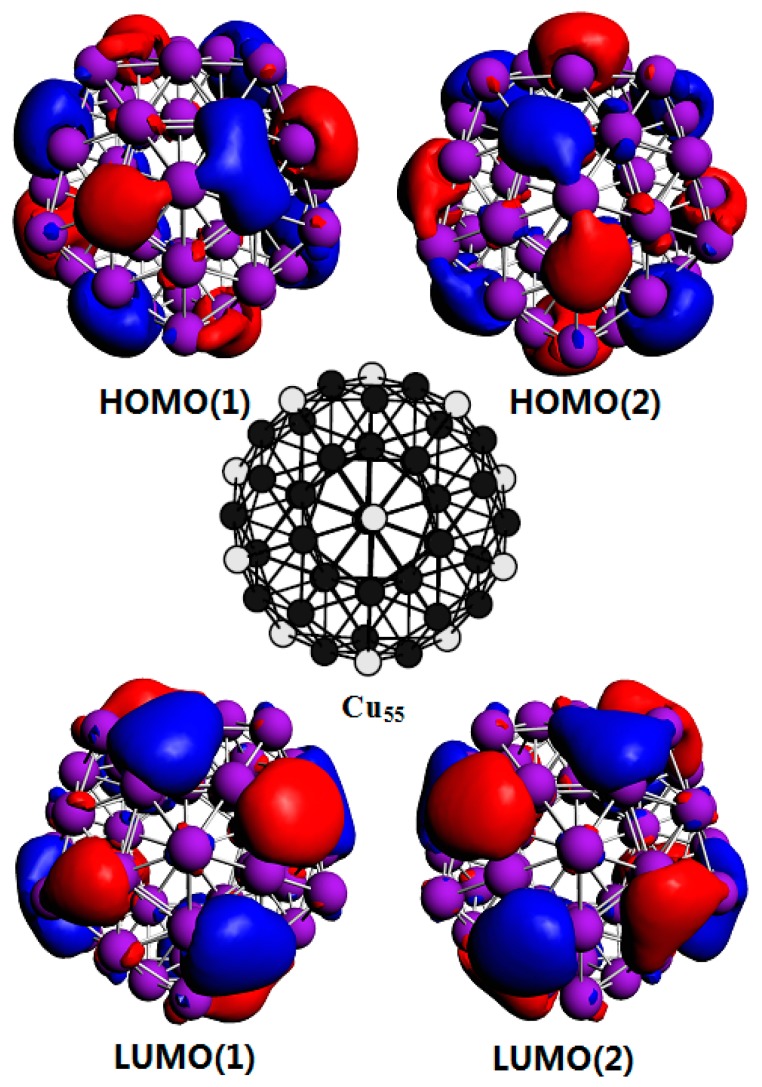
The top part shows the HOMO(1) and HOMO(2) corresponding to two degenerate highest occupied molecular orbitals (HOMO). The bottom part shows the LUMO(1) and LUMO(2) referring to two degenerate lowest unoccupied molecular orbitals (LUMO). The middle structure diagram is the ground state structure of Cu_55_ cluster, in which white balls represent equivalent atoms.

As mentioned above, we have demonstrated that the interaction between two Cu clusters is anisotropic. In order to reveal the origin of the anisotropic interaction between two Cu clusters, the aggregation of two separated Cu_55_ clusters to Cu_110_(a) and Cu_110_(d) have been investigated. For the convenience of discussion, the Cu atoms in the Cu_55_ cluster are divided into two types. The equivalent atoms which can exchange positions by symmetry operation are represented with white in Figure 4, the other atoms are represented with black. Our results indicate that other atoms or clusters are hard to adsorb on the tops (top sites) of the white atoms. In some cases, other atoms can be adsorbed; however its adsorption energy is relatively low, leading to unstable structures. For example, in the case of Cu_110_(a) shown in Figure 3a, two Cu_55_ clusters are adsorbed on the top of each white atom but the binding energy is less than 1.0 eV. On the other hand, it is more favorable for other atoms or clusters to adsorbed on the hollow site consisting of three adjacent black atoms. In the initial structure of Cu_110_(b) shown in Figure 3a, a Cu_55_ cluster is adsorbed on the hollow site of another Cu_55_ cluster. Consequently, the binding energy of Cu_110_(d) is 7.5 eV higher that of Cu_110_(b), indicating that Cu_110_(d) is more stable than Cu_110_(b).

Figure 4 shows the highest occupied molecular orbitals (HOMO) and lowest unoccupied molecular orbitals (LUMO) of the Cu_55_ cluster. Both of them are degenerate orbitals. It is found that the electron density distribution of the HOMO is mainly concentrated on the hollow sites, while the charge density distribution on the top sites is very low. This feature is more pronounced for the LUMO of Cu_55_ cluster. This is the main reason why Cu clusters are easily adsorbed on hollow sites. The most stable adsorption position that can attract other clusters differs from cluster to cluster. The compact Cu_44_(b) shown in Figure 3b is still a meta-stable structure. Our calculation results show that some of hollow sites are not energy favorable for the adsorption of other clusters, as shown in Cu_88_(a). On the other hand, some of its top sites can strongly attract other clusters as shown in Cu_88_(c). This further complicates the aggregation of Cu clusters. However, by checking the HOMO and LUMO (not shown here) of Cu_44_(b) clusters, it is clear that the charge density distribution of the HOMO and LUMO of Cu_44_(b) are mainly concentrated on top sites, which indicates that the charge distribution of Cu clusters is of vital importance in determining the aggregation of Cu clusters. Therefore, the strong interaction positions depend mainly on the charge distribution rather than on geometry positions.

### 3.4. Simulation of the Aggregation of Copper Clusters on a Liquid Surface

**Figure 5:**
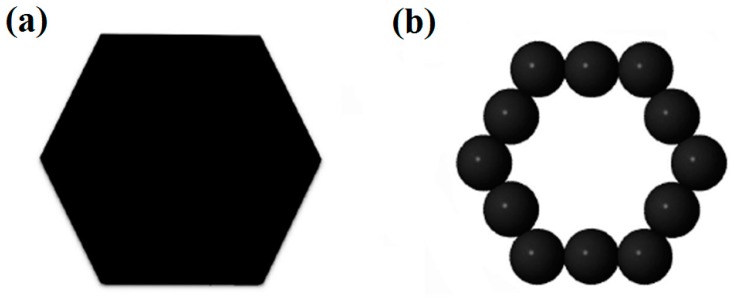
In a two-dimension system: A (**a**) hexagon-shaped cluster is treated as a (**b**) sphere-connected model.

**Figure 6:**
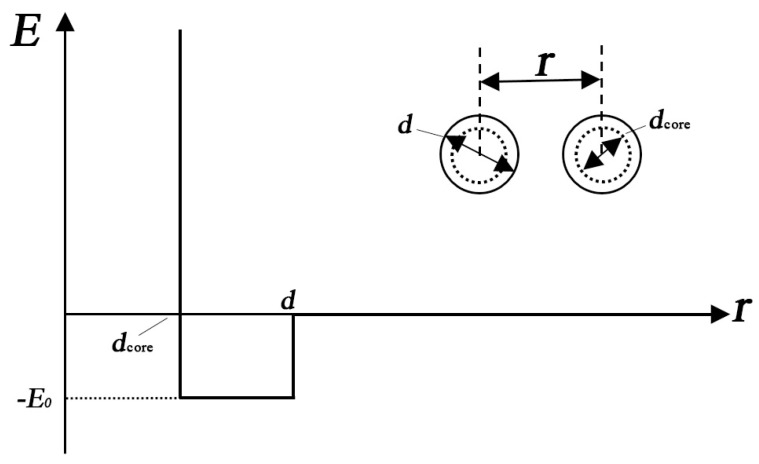
The potential well between two spheres belonging to two different clusters.

**Figure 7:**
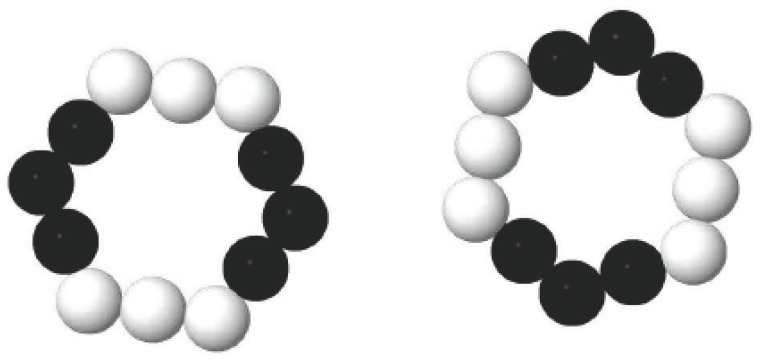
Sides of the hexagons are assigned to be different in order to study their anisotropic properties.

For geometric configurations of copper clusters deposited on a liquid surface, a two-dimensional polygons system has been constructed based on our simulation results. As we have discussed above, most stable Cu clusters are constructed by hexagons and pentagons structures as shown in Figure 1. In some cases, triangles, squares and heptagons can also be observed. During MC simulations, the simulation results are nearly the same when triangles, squares, pentagons, hexagons or heptagons are chosen to model Cu clusters. Therefore, in the present study, hexagons (Figure 5a) have been chosen during the MC simulation. However, the hexagons cannot be treated as the basic units in simulations. The common strategy is to treat each side of a hexagon as *n* linearly-connected spheres [39]. Figure 5b shows a small hexagon with *n* = 3 and a total number of *N* =12 for spheres. Larger hexagons with more spheres on each side have been also used to simulate the aggregation of Cu clusters but the results do not change substantially. By assuming that there is no interaction between two clusters until they touch each other, the interaction between two spheres from surrounded clusters can be simplified as a potential well as shown in Figure 6. When the center-center distance *r* between two spheres is within the range from *d_core_* to *d*, they attract each other due to the potential well. However, when the hard-core of spheres overlap (the center-center distance *r* is smaller than *d_core_*), the infinitely repulsive force dominates. Conversely, when the distance is larger than *d*, the interaction between them can be neglected. Moreover, on the basis of anisotropic interaction between two Cu clusters which have been discussed above, the binding energy of each side is set to be different when these hexagons combine with each other during the simulation. The cluster is assigned to a hexagonal shape with each side has 3 spheres. In order to study the anisotropic interactions between two clusters, we assign two diagonal sides of a hexagon (the white spheres in Figure 7) to be different from the other sides (black spheres). 

Considering that the maximum ratio of binding energies between two clusters in different directions is within the range from 7 to 15, the binding energy ratio of 9 is chosen during our simulations. It is worth noting that choosing different binding energy ratio from 7 to 15 only affects the lengths of cluster aggregations but the morphology of Cu clusters aggregation does not change. Therefore, when the distance between two white spheres lies in the attraction range, there is a drop of −0.9 E_0_ (a binding energy between two clusters.) and the value is −0.1 E_0_ for the interaction between a white sphere and a black sphere. The attraction between two black spheres is assumed to be zero. 

**Figure 8:**
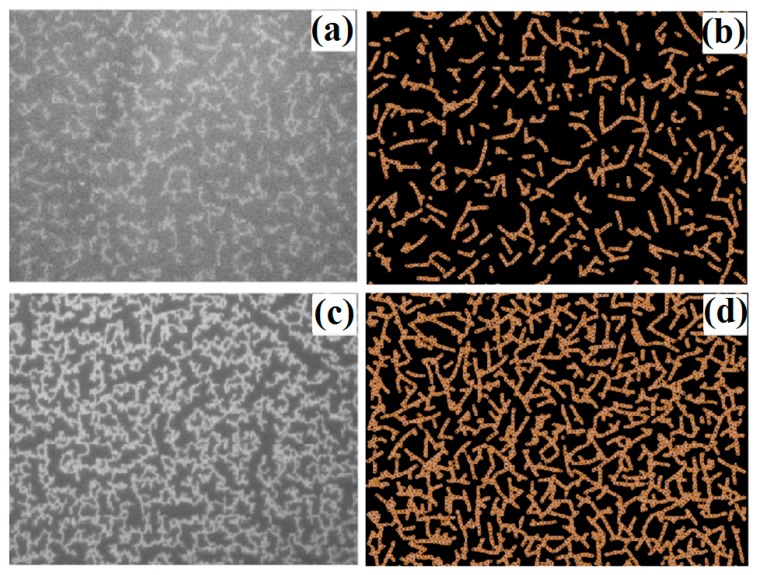
(**a,c**) are the typical experimental images of the Cu atomic aggregates on silicone oil surfaces [23]. (**b**,**d**) are the simulation results obtained by the MC method.

Figure 8a,c present the typical experimental images of the Cu atomic aggregates on silicone oil surfaces obtained by our group previously [23]. Their nominal film thickness is 1.0 and 5.0 nm, respectively. In the experiment, the deposition rate is f = 0.08 nm/s. Each image has a size of 56 × 42 µm^2^. We run the simulation on a 2-dimensional scale of 400 d × 400 d by means of MC method. Figure 8b,d show the simulation results obtained by the above method. They correspond to the condensation of 2000 and 4000 clusters on the liquid surface, respectively. The calculation shows that the fractal dimensions of Figure 8c,d are 1.87 and 1.83, respectively. This further indicates that the simulation results are in good agreement with the experimental results.

## 4. Conclusions

Using the ADF and ATK programs based on first-principles calculations, we have investigated the structures and aggregates of the small or medium-sized Cu clusters. Their aggregation on silicone oil surfaces is simulated by our self-programming and some conclusions are drawn. 

1. Most of the Cu clusters adopt icosahedral structures, which can be derived from the 13-atom icosahedron. Some clusters with *n* = 5, 8, 18, 20, 26, 28 are more stable than their neighboring clusters within the scope of our global search. 

2. The aggregation of the Cu clusters depends on their structures and directions. In some directions, they converge tightly but in other directions, the attractions between them are very weak. The HOMO and LUMO analyses reveal that this anisotropic interaction between Cu clusters is determined by the charge distribution of the clusters. 

3. The aggregation of the copper clusters on a liquid surface has been simulated by means of MC method. The fractal dimension with 4000 clusters is 1.83, which is close to the experimental value 1.87 with nominal film thickness d_f_ = 5 nm. The simulation results of the morphologies are in good agreement with the experimental results.

## Figures and Tables

**Table 1:** The binding energies (BE, in eV) of Cu_n_ (*n* =2–80) clusters obtained by the Amsterdam Density Functional (ADF) and Atomistix ToolKit (ATK) programs, respectively. The data marked with * in the upper right corner is calculated from the experimental data [19,39]. The newly revealed structures are marked by the letter “a”. The letter “b” is used to refer to the structures in the previous study. The first and second lines of the table correspond to the data calculated by ADF and ATK respectively.

Cu_n_	BE	Cu_n_	BE	Cu_n_	BE	Cu_n_	BE	Cu_n_	BE
Cu_2_	2.47	Cu_16_	35.17	Cu_29_	68.08	Cu_45_	111.02	Cu_63_	160.52
	2.58		41.79		81.32		133.22		193.04
Cu_3_	3.69	Cu_17_	38.18	Cu_30_	71.09	Cu_46_	114.81	Cu_64_	163.45
	4.00		45.23		85.05		137.89		196.63
	3.21 *	Cu_18_	40.98	Cu_31_(a)	74.11	Cu_47_	117.46	Cu_65_	165.98
Cu_4_	4.88		48.47		88.58		141.03		199.82
	5.68	Cu_19_	43.14	Cu_31_(b)	74.14	Cu_48_	119.50	Cu_66_	169.07
	5.92 *		51.21		88.52		143.53		203.19
Cu_5_	7.89	Cu_20_	45.89	Cu_32_	76.82	Cu_49_	123.33	Cu_67_	171.52
	9.00		54.40		91.83		148.18		206.57
	7.80 *	Cu_21_	48.26	Cu_33_	79.67	Cu_50_	125.15	Cu_68_	174.76
Cu_6_	9.91		57.29		95.14		150.33		209.99
	11.80	Cu_22_	50.32	Cu_34_	82.36	Cu_51_	127.93	Cu_69_	176.76
	10.38 *		59.91		98.26		154.00		212.87
Cu_7_	13.40	Cu_23_(a)	53.14	Cu_35_	85.26	Cu_52_	131.33	Cu_70_	179.57
	15.33		63.30		101.83		157.99		216.22
	13.02 *	Cu_23_(b)	52.80	Cu_36_	87.58	Cu_53_	134.57	Cu_71_	182.38
Cu_8_	16.06		63.04		104.81		161.90		219.77
	18.47	Cu_24_(a)	55.62	Cu_37_	90.26	Cu_54_	137.97	Cu_72_	185.20
	16.00 *		66.07		108.04		165.85		223.11
Cu_9_	17.89	Cu_24_(b)	55.00	Cu_38_	93.08	Cu_55_	141.24	Cu_73_	187.97
	20.77		65.63		111.53		169.80		226.41
Cu_10_	19.83	Cu_25_(a)	57.77	Cu_39_	95.66	Cu_56_	143.81	Cu_74_	190.55
	23.45		69.01		114.50		172.75		229.72
Cu_11_	22.02	Cu_25_(b)	57.35	Cu_40_	98.53	Cu_57_	146.41	Cu_75_	193.34
	26.05		68.56		118.01		175.73		233.20
Cu_12_	24.36	Cu_26_(a)	60.66	Cu_41_	100.45	Cu_58_	149.39	Cu_76_	196.20
	28.76		72.33		120.31		179.14		236.56
Cu_12_	24.38	Cu_26_(b)	59.68	Cu_42_	104.06	Cu_59_	150.69	Cu_77_	199.21
	28.78		71.64		124.59		180.85		240.04
Cu_13_	26.32	Cu_27_	62.62	Cu_43_(a)	106.50	Cu_60_	154.45	Cu_78_	202.35
	31.58		74.90		127.66		185.30		243.34
Cu_14_	29.19	Cu_28_(a)	65.83	Cu_43_(b)	104.87	Cu_61_	157.05	Cu_79_	204.59
	34.88		78.41		127.66		188.65		246.44
Cu_15_	32.21	Cu_28_(b)	65.42	Cu_44_	109.00	Cu_62_	159.56	Cu_80_	207.37
	38.38		78.24		130.74		191.59		249.72
